# Chemical Profiling and Antimicrobial Activity of the Leaf Essential Oil of *Vepris nobilis* (Delile) Mziray: In Silico Evaluation of the Major Constituent, Germacrene D

**DOI:** 10.3390/ijms27177927

**Published:** 2026-09-05

**Authors:** Biniam Paulos, Mariamawit Y. Yeshak, Avijit Mazumder, Peter Lindemann, Daniel Bisrat, Kaleab Asres

**Affiliations:** 1 Department of Pharmaceutical Chemistry and Pharmacognosy, School of Pharmacy, College of Health Sciences, Addis Ababa University, Addis Ababa P.O. Box 1176, Ethiopia; biniam.paulos@aau.edu.et (B.P.); mariamawit.yonathan@aau.edu.et (M.Y.Y.); daniel.bisrat@aau.edu.et (D.B.); 2 Department of Pharmacology, Noida Institute of Engineering and Technology (Pharmacy Institute), 19 Knowledge Park II, Institutional Area, Greater Noida 201306, India; avijitmazum@yahoo.com; 3 Division Pharmaceutical Biology and Pharmacology, Institute of Pharmacy, Martin-Luther-University, Halle-Wittenberg, Hoher Weg 8, D-06120 Halle, Germany; drpeterlindemann@online.de

**Keywords:** *Vepris nobilis*, essential oil, chemical composition, germacrene D, antimicrobial activity, molecular docking

## Abstract

Antimicrobial resistance is a growing global health challenge, highlighting the need for new bioactive compounds from medicinal plants. *Vepris nobilis* is traditionally used in East Africa for treating infections and respiratory disorders; however, its essential oil (EO) composition and antimicrobial mechanisms remain poorly characterized. This study investigated the chemical composition and antimicrobial activity of *V. nobilis* EO, along with an in silico evaluation of its major constituent, germacrene D. The EO was extracted by hydrodistillation and analyzed using gas chromatography-mass spectrometry (GC–MS). Its antimicrobial activity was evaluated against 26 bacterial and 4 fungal strains using disc diffusion, broth microdilution, and MBC/MFC (Minimum Bactericidal Concentration/Minimum Fungicidal Concentration) assays. Germacrene D showed stronger activity than the EO, particularly against both multidrug resistant (MDR) and non-MDR Gram-negative bacterial strains (MIC = 10 µg/mL), with bactericidal and fungicidal effect. Molecular docking of germacrene D against two clinically relevant enzymes—dehydrosqualene synthase (CrtM) from *Staphylococcus aureus* and SWISS-modeled sterol 14-α-demethylase (CYP51) from *Penicillium funiculosum*—suggested potential interactions with both targets, with a favorable predicted binding affinity for CrtM (−7.654 kcal/mol) and for CYP51 (−5.898 kcal/mol). These findings provide preliminary insights into a possible antimicrobial mechanism, although experimental validation is needed to confirm this hypothesis. ADMET analysis suggested favorable drug-like properties despite limited solubility. These findings provide scientific support for the traditional use of *V. nobilis* leaves in the treatment of respiratory infections and highlight germacrene D as a promising lead compound for further antimicrobial development.

## 1. Introduction

Essential oils (EOs) are complex; volatile mixtures of secondary metabolites produced by aromatic plants and have been used for centuries in traditional medicine for the prevention and treatment of various infectious and other diseases. Their broad-spectrum activity against pathogenic bacteria and fungi has attracted considerable scientific interest, particularly in the context of the growing global burden of antimicrobial resistance (AMR). The emergence and dissemination of drug-resistant microorganisms, together with the declining effectiveness of some conventional antimicrobial agents and concerns regarding their adverse effects, have intensified the search for new, effective, and relatively safe antimicrobial agents from natural sources. Plant-derived EOs represent a particularly promising reservoir because of their chemical diversity and ability to act on multiple microbial targets [1].

The antimicrobial activity of EOs is largely determined by their complex chemical composition, which may include monoterpenes, sesquiterpenes, phenols, alcohols, aldehydes, ketones, esters, and other volatile constituents. The biological activity of an EO is not necessarily attributable to a single compound but may result from the combined effects of its constituents, including additive, synergistic, or antagonistic interactions. Among these constituents, oxygenated monoterpenes and phenolic compounds such as thymol, carvacrol, and eugenol are frequently associated with pronounced antimicrobial activity. However, hydrocarbon terpenes and sesquiterpenes can also contribute substantially to the overall activity of an EO. Moreover, the chemical profile and biological activity of an EO may vary considerably according to plant species, geographical origin, developmental stage, environmental conditions, plant part, and extraction method [2]. Consequently, characterization of both the chemical composition and antimicrobial activity is essential when evaluating medicinal plants as potential sources of antimicrobial agents. EOs exert antibacterial and antifungal effects through several, often overlapping, mechanisms. Their lipophilic constituents can interact with microbial cell envelopes and partition into cytoplasmic membranes, resulting in altered membrane fluidity and permeability, leakage of intracellular constituents, disruption of ion gradients, and loss of cellular homeostasis. These effects may subsequently impair ATP generation, membrane-associated enzymes, and other essential metabolic processes, ultimately leading to growth inhibition or cell death [1]. In fungi, EOs may additionally interfere with membrane sterols, particularly ergosterol, and affect cell-wall-associated processes and other cellular pathways [3]. The ability of EOs and their constituents to affect multiple cellular targets has generated considerable interest in their potential to complement conventional antimicrobials and possibly reduce the likelihood of resistance development. However, the precise contribution of individual EO constituents to antimicrobial activity and the interactions among constituents remain insufficiently understood.

An important consideration in evaluating EOs is therefore the relationship between chemical abundance and biological activity. The major constituent of an EO is not necessarily its principal antimicrobial determinant, because minor constituents may enhance, diminish, or otherwise modify the activity of the major component. Conversely, an apparently weakly active EO may contain a highly active constituent whose activity is influenced by the physicochemical environment of the complete oil. Investigating the activity of both the whole EO and its major constituent can therefore provide valuable insight into the contribution of individual compounds and possible interactions within the complex mixture [3]. Molecular docking and in silico ADMET analyses can complement experimental antimicrobial data by providing preliminary insights into potential molecular targets, ligand-target interactions, as well as predicted pharmacokinetic and toxicology related properties [4,5]. Nevertheless, computational approaches should be considered supportive evidence rather than definitive proof of antimicrobial mechanism.

Plants of the genus *Vepris* (Rutaceae) are particularly interesting in this regard because several species have been traditionally used in African ethnomedicine and have yielded chemically diverse secondary metabolites and EOs [6,7]. *Vepris nobilis* (Delile) Mziray (syn. *Teclea nobilis* Delile) has attracted considerable phytochemical interest, with furoquinoline alkaloids and other secondary metabolites reported from different plant parts [8]. The leaf essential oil has also been previously subjected to chemical and biological evaluation [9]. In addition, the leaves have traditionally been used in East Africa for the management of respiratory tract infections, including pneumonia, through inhalation of the smoke generated by burning the leaves [10].

Such ethnomedicinal use provides a valuable basis for investigating its potential anti-infective properties. However, despite this traditional application, the chemical composition and antimicrobial potential of the leaf EO of *V. nobilis*, particularly against clinically relevant bacterial and fungal pathogens, remain insufficiently characterized. Furthermore, the relationship between its major chemical constituent(s) and the antimicrobial activity of the whole EO has not been adequately investigated, and information regarding their possible molecular interactions and predicted pharmacokinetic and safety characteristics is limited.

Therefore, the present study was designed to address these gaps by characterizing the chemical composition of the leaf EO of *V. nobilis*, evaluating its antibacterial and antifungal activities, comparing the antimicrobial activity of the EO with that of its major constituent, and investigating the possible molecular basis of the activity using molecular docking. In addition, in silico ADMET analysis was performed to obtain preliminary information on the pharmacokinetic and safety profiles of the major constituent. By integrating ethnomedicinal evidence, chemical profiling, antimicrobial evaluation, and computational analyses, this study seeks to provide a scientific basis for the traditional use of *V. nobilis* and to assess its potential as a source of bioactive compounds for antimicrobial drug.

## 2. Results

### 2.1. Essential Oil Composition

Hydrodistillation of the air-dried leaves of *V. nobilis* yielded 0.8% (*v*/*w*) of essential oil. GC-MS analysis identified 37 compounds representing 87.48% of the total oil composition of *V. nobilis* (Table 1). Sesquiterpenes accounted for 71.85% of the total oil, with germacrene D alone representing 64.75%. In addition, appreciable amounts of sesquiterpenoids such as guaiol acetate (3.48%), and τ-cadinol (2.38%) were detected (Figure S1).

### 2.2. In Vitro Antibacterial Activity

The antibacterial activities of the EO from *V. nobilis* as well as its major constituent, germacrene D, were evaluated using both the agar disc diffusion method and broth microdilution assay. The results of these tests provided complementary information regarding the antibacterial properties of the plant’s EO and its major constituent (Table 2). The EO of *V. nobilis* had moderate antibacterial activity against the tested bacterial strains, producing inhibition zones ranging from 8.7 to 17 mm in diameter at a concentration of 200 µg/mL, with minimum inhibitory concentration (MIC) values of 10–400 µg/mL. In contrast, germacrene D, the major sesquiterpene constituent of the EO, exhibited moderate antibacterial activity, with inhibition zones ranging from 9.5 to 19 mm and MIC values ranging from 10 to 50 µg/mL.

Germacrene D exhibited notable antibacterial activity against *S. aureus* ML 267, a Gram-positive bacterium, with an inhibition zone of 19.0 ± 0.5 mm and an MIC of 50 µg/mL. Importantly, its activity against the MDR strains (*S. aureus* MDR 1, *S. aureus* MDR 2, *E. coli* C600, *E. coli* HB101, and *P. aeruginosa* MDR 1) was particularly pronounced, with a relatively low MIC of 10 µg/mL.

### 2.3. In Vitro Antifungal Activity

Clear differences among the tested samples were evident based on both the ZOI and MIC values (Table 3). Although griseofulvin produced the largest inhibition zones against *Aspergillus niger* and *Candida albicans*, indicating efficient diffusion through the agar medium, its relatively high MIC values (400–500 µg/mL) suggest lower intrinsic antifungal potency. In contrast, germacrene D exhibited a substantially lower MIC (50–100 µg/mL), indicating greater antifungal potency despite producing smaller inhibition zones against these two fungal strains.

The EO demonstrated the highest zone of inhibition (14.0 mm) as compared to both germacrene D and griseofulvin. However, the higher MIC of the EO (400 µg/mL) implies that more amount is required to inhibit fungi completely. Nonetheless, germacrene D showed inhibition zones of 13.0–14.5 mm, with MIC values of 50–100 µg/mL.

### 2.4. Mode of Action

Germacrene D exhibited bactericidal and fungicidal activities at concentrations of 200 µg/mL against bacteria and 1500 µg/mL against fungi. However, against *Bacillus pumilus* and *B. subtilis*, it displayed bacteriostatic rather than bactericidal activity.

### 2.5. In Silico Analysis

#### 2.5.1. Homology-Modeled Structure of Sterol 14-α-Demethylase

Since the three-dimensional (3D) structure of the potential antifungal target, sterol 14-α-demethylase (CYP51) from *P. funiculosum*, was not experimentally available, a homology modeling approach was employed using the SWISS-MODEL Workspace to construct its structural model. The protein sequence from *P. notatum* (UniProt accession ID: A0ABQ8W6D1; 515 amino acids) was used to construct a homology model of sterol 14α-demethylase (CYP51) from *P. funiculosum*. The sequence was successfully aligned with a suitable template, resulting in a highly reliable and high-quality three-dimensional structural model.

The quality and reliability of the generated model were rigorously assessed using multiple validation metrics, as summarized in Table 4. The Global Model Quality Estimate (GMQE) score, which reflects both the template–target alignment and the template quality, was found to be 0.92, indicating excellent model reliability. This was further corroborated by a high sequence identity of 93.59% between the target and the selected template, suggesting a strong evolutionary relationship and high structural conservation.

The homology modeling approach was selected over other structure prediction methods due to the exceptionally high sequence identity (93.59%) between the target CYP51 sequence and the available template, which ensures high structural reliability. The robust validation metrics obtained for the model (GMQE 0.92; QMEANDisCo 0.81 ± 0.05; 96.88% residues in favored Ramachandran regions) confirm that the generated structure is of high quality and suitable for molecular docking studies.

In addition, the absolute quality of the model was evaluated using the QMEAN scoring function. The overall QMEAN score was 0.43, which, while moderate, falls within an acceptable range for computational structural studies and reflects a reasonable agreement between the model and expected physical properties. The QMEANDisCo global score, which provides a per-residue estimate of model confidence, was calculated as 0.81 ± 0.05, indicating high local accuracy and strong coordination between residues throughout the structure.

**Figure 1:**
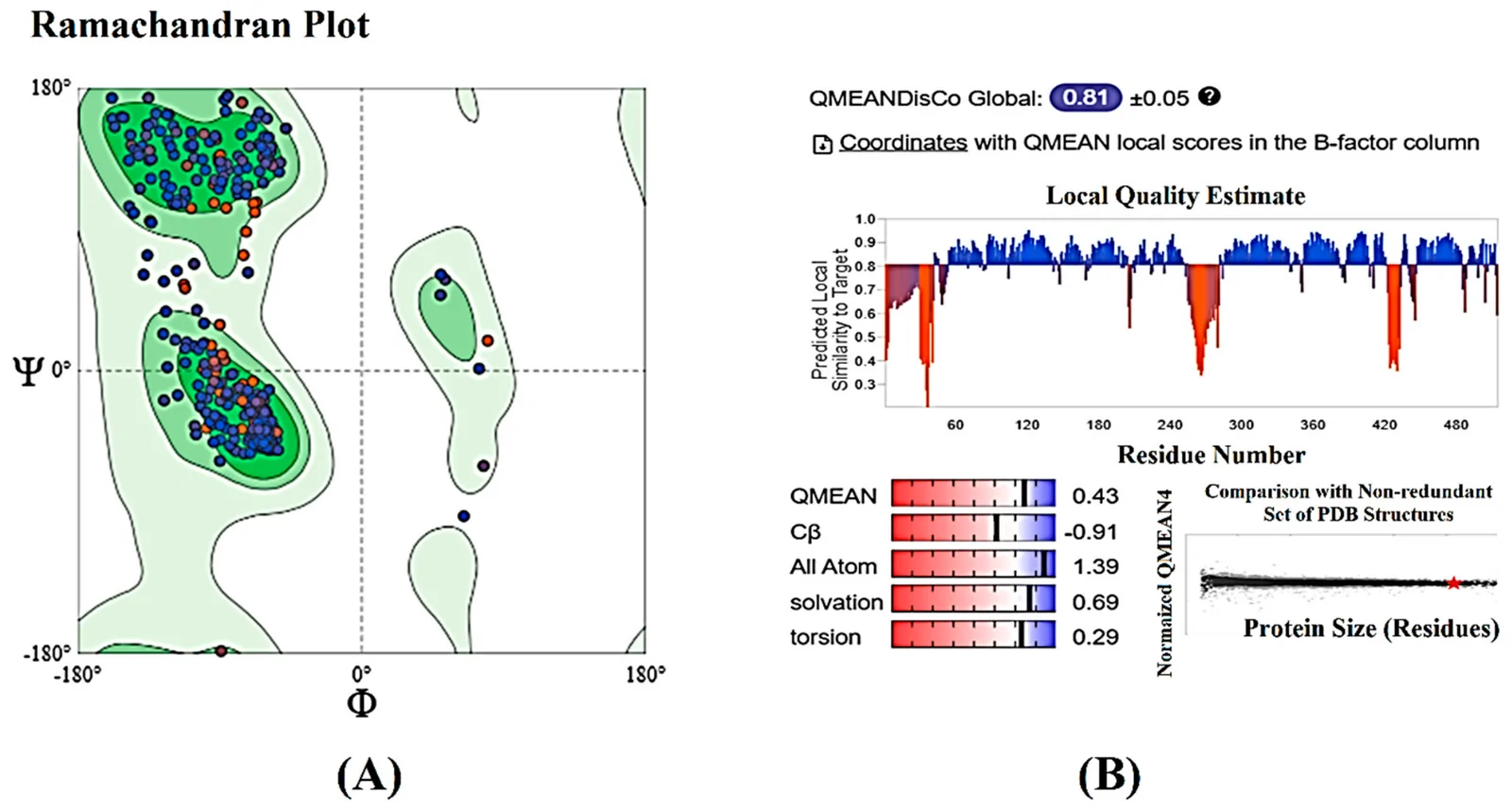
Validation of the homology-modeled CYP51 structure from *Penicillium funiculosum* using stereochemical and global quality estimation tools. (**A**) Ramachandran plot showing residue conformations; (**B**) QMEANDisCo global score plot illustrating per-residue model confidence.

To further validate the stereochemical quality of the model, a Ramachandran plot analysis was performed. The results revealed that 96.88% of all amino acid residues were located in the most favored regions, with no residues in disallowed conformations (Figure 1). This high percentage confirms the excellent stereochemical integrity of the modeled structure and supports its suitability for downstream in silico studies, such as molecular docking and virtual screening for antifungal drug discovery.

Overall, the combination of high GMQE and sequence identity, acceptable QMEAN scores, and outstanding Ramachandran statistics demonstrates that the generated CYP51 model is robust, reliable, and structurally sound for future computational analyses.

#### 2.5.2. Molecular Docking Predictions

Molecular docking was used to predict the binding modes and affinities of germacrene D toward two clinically relevant targets: dehydrosqualene synthase (CrtM) from *Staphylococcus aureus* and homology-modeled sterol 14α-demethylase (CYP51) from *P. funiculosum*. CrtM was selected based on its hydrophobic active site and role in isoprenoid biosynthesis, which supports its interaction with hydrophobic sesquiterpenes [11]. CYP51 was selected based on previous docking studies demonstrating interactions of mono- and sesquiterpene hydrocarbons with its hydrophobic binding cavity, supporting its potential relevance to the antifungal activity of germacrene D [12].

##### Molecular Docking Against the Antibacterial Target Dehydrosqualene Synthase (CrtM) of *Staphylococcus aureus*

To evaluate the potential antibacterial activity of germacrene D, molecular docking studies were performed against dehydrosqualene synthase (CrtM) from *S. aureus* (PDB ID: 4F6V), a key enzyme in the staphyloxanthin biosynthetic pathway. Staphyloxanthin is a carotenoid pigment that contributes to the virulence and oxidative stress resistance of *S. aureus*, making CrtM an attractive target for antivirulence therapy. The docking simulations were conducted using the Glide standard precision (SP) module of Schrödinger Suite 25.2, with the co-crystallized native ligand (ZYM) serving as a reference for binding site identification and comparative analysis.

The docking results are summarized in Table 5, demonstrating that germacrene D exhibited a favorable binding affinity toward the CrtM active site, with a Glide score (G.Score) of −7.654 kcal/mol. Although this score was lower than that of the native ligand (−12.265 kcal/mol), it still indicates a moderately strong and energetically favorable interaction, suggesting that germacrene D may act as a competitive inhibitor of the enzyme.

**Figure 2:**
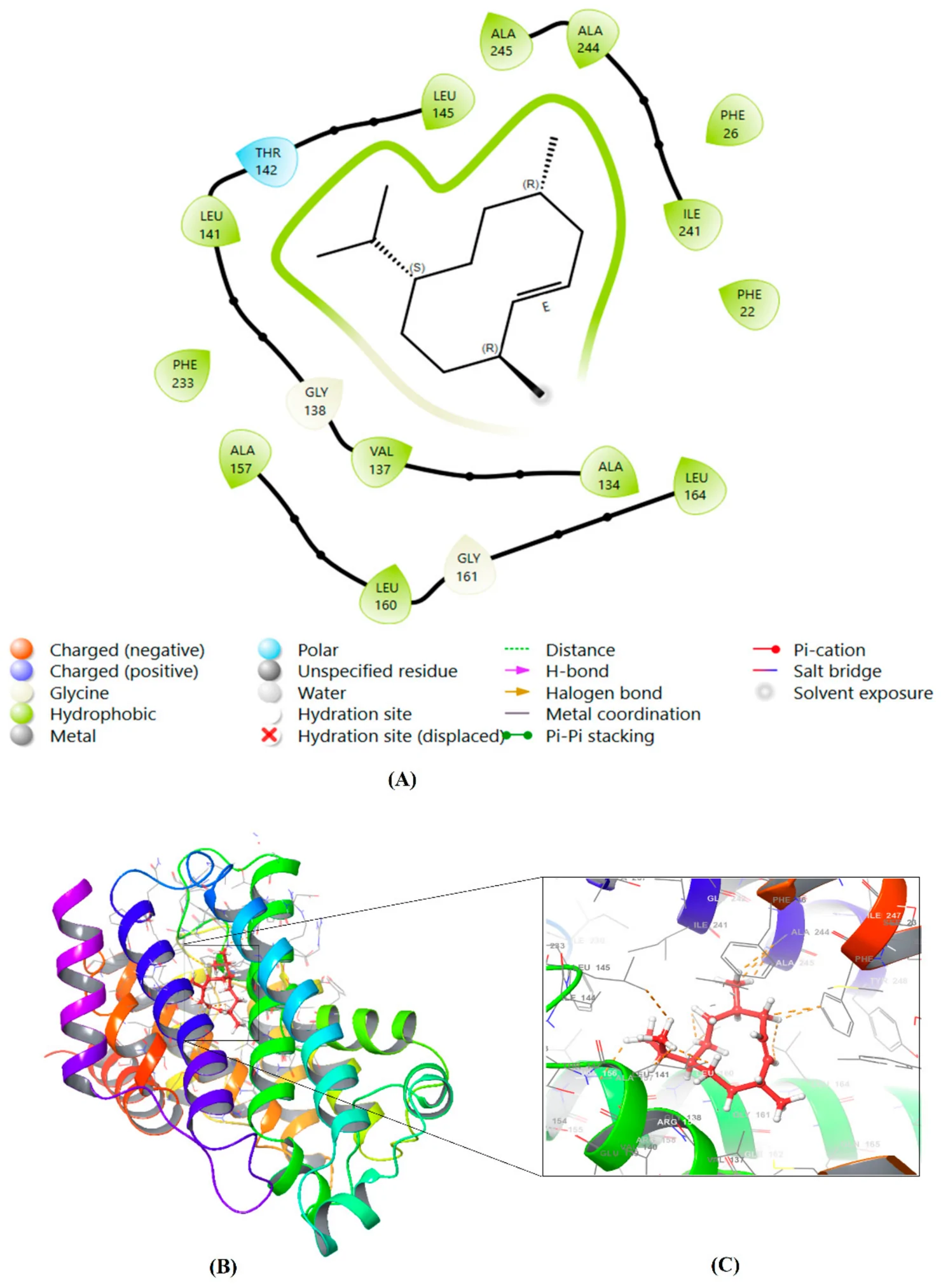
Molecular docking analysis of germacrene D in the catalytic binding pocket of *Staphylococcus aureus* dehydrosqualene synthase (CrtM). (**A**) 2D interaction diagram. (**B**) Overall 3D binding conformation. (**C**) Zoomed-in view of the 3D interaction conformation.

Detailed analysis of the protein–ligand complex for germacrene D showed that its binding was predominantly stabilized by extensive hydrophobic interactions with several key residues lining the active-site cavity. These included ALA134, ALA244, ALA245, ILE241, LEU141, LEU145, LEU160, LEU164, PHE22, PHE26, PHE233 and VAL137. The high proportion of nonpolar contacts suggests that hydrophobic forces play a major role in anchoring germacrene D within the binding pocket. In addition to these hydrophobic interactions, a polar interaction was observed with THR142, which may contribute modestly to binding specificity and orientation (Figure 2).

In contrast, the native ligand ZYM (2,4-dioxo-4-{[3-(3-phenoxyphenyl)propyl] amino}butanoic acid) demonstrated a substantially higher binding affinity, with a Glide Score of −12.265 kcal/mol. This superior binding energy can be attributed to a combination of multiple strong interaction types, including: π–π stacking between the aromatic rings of ZYM and TYR141; Hydrogen bonding with key polar residues, namely ASP48, ASN168, and ARG265; Extensive hydrophobic contacts with residues such as MET15, PHE22, PHE26, VAL37; ILE40, ARG45, and others (Table 5). In addition, the root-mean-square deviation (RMSD) between the docked pose and the native ligand was within acceptable limits, confirming the reliability of the docking protocol.

##### Molecular Docking Against the Homology-Modeled Sterol 14α-Demethylase (CYP51) of *Penicillium funiculosum*

To assess the potential antifungal activity of germacrene D, molecular docking studies were further performed against homology-modeled sterol 14-α-demethylase (CYP51) from *P. funiculosum*, using the homology-modeled structure described in Section 2.5.1. The docking simulations were conducted using the Glide standard precision (SP) module of Schrödinger Suite 25.2, with the active site defined based on the conserved heme-binding region and substrate-access channel characteristic of the CYP51 family.

As summarized in Table 6, germacrene D exhibited a moderate binding affinity toward the homology-modeled structure of *P. funiculosum* CYP51 enzyme, with a Glide score (G.Score) of −5.898 kcal/mol. Detailed analysis of the protein–ligand interaction profile for germacrene D revealed that its binding was stabilized by a combination of polar and hydrophobic contacts. Specifically, polar interactions were observed with three key residues: THR116, THR300, and HIE307 (histidine 307). Among these, HIE307 is of particular significance, as it corresponds to the conserved histidine residue that coordinates with the heme iron in CYP51 enzymes—a critical interaction for azole-based antifungals. The polar contacts with THR116 and THR300 may further contribute to proper ligand orientation within the binding pocket.

**Figure 3:**
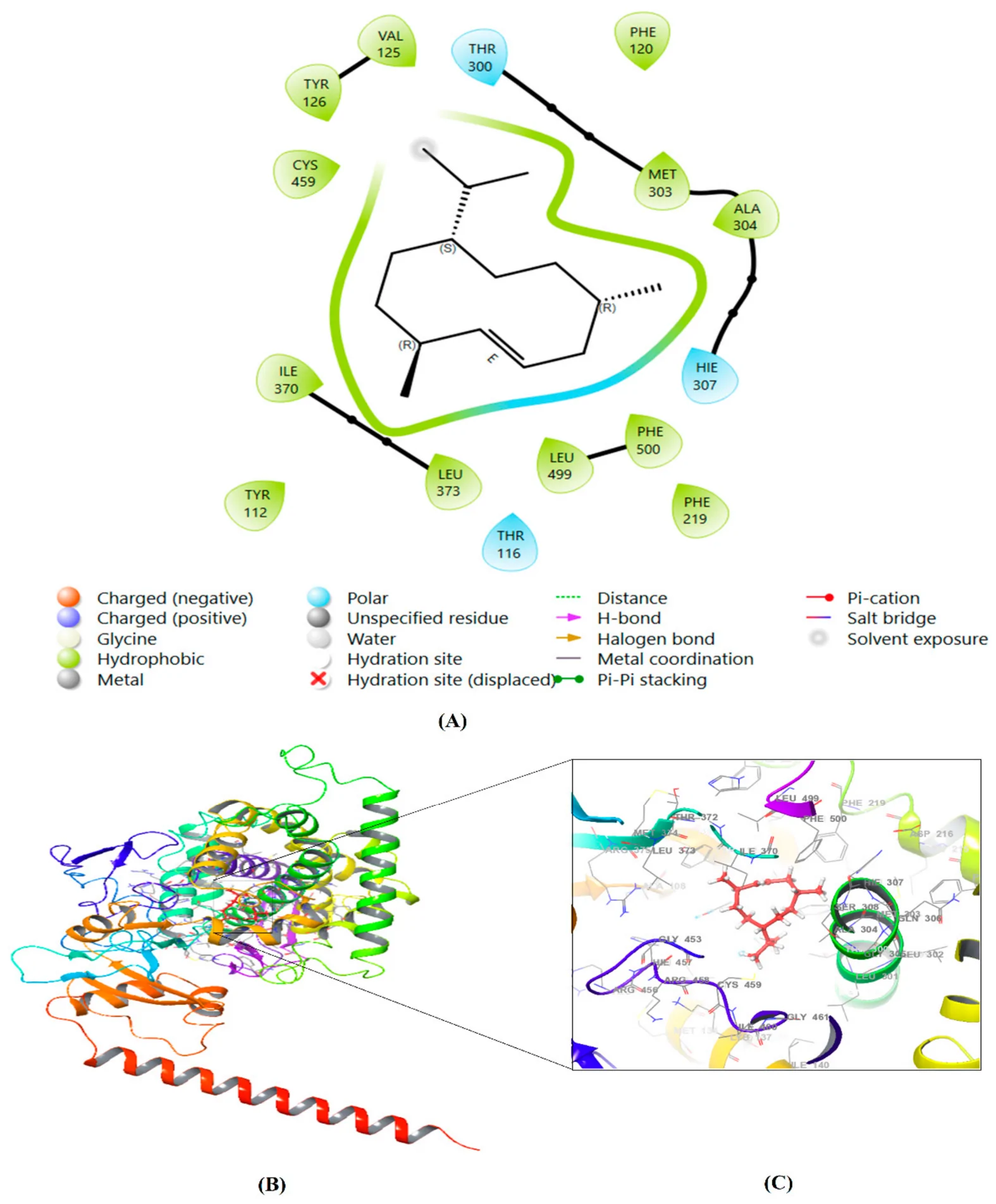
Molecular docking analysis of germacrene D within the catalytic binding pocket of the homology-modeled sterol 14α-demethylase (CYP51) from *Penicillium funiculosum*. (**A**) 2D interaction diagram. (**B**) Overall 3D binding conformation. (**C**) Zoomed-in view of the 3D interaction conformation.

In addition to these polar interactions, the remaining interacting residues were predominantly hydrophobic in nature, including ALA304, CYS459, ILE370, LEU373, LEU499, MET303, PHE219, PHE500, TYR112, TYR126 and VAL125 (Figure 3). This extensive network of hydrophobic contacts suggests that germacrene D is well embedded within the nonpolar core of the CYP51 active site, which is lined with aromatic and aliphatic residues that facilitate stable binding through van der Waals forces and π–alkyl interactions. The presence of multiple phenylalanine residues (PHE120, PHE219, PHE500) may also allow for potential π–π stacking interactions with the diene system of germacrene D, although these were not explicitly identified in the docking output.

Overall, these in silico findings indicate that germacrene D possesses moderate antifungal potential through its interaction with CYP51. While its binding affinity is not as strong as that of established azole drugs, the compound’s unique interaction profile—dominated by hydrophobic contacts with key active-site residues—provides a valuable starting point for lead optimization.

### 2.6. Drug-Likeness and Pharmacokinetic Studies

According to the QikProp prediction, germacrene D exhibited favorable physicochemical drug-likeness (Table 7). It has an appropriate molecular weight (<500 g/mol). In addition, the compound satisfied the criteria of Lipinski’s rule of five, thus being considered a good oral drug candidate. The predicted lipophilicity was within the desired range, thereby ensuring efficient permeability across biological membranes; however, the predicted solubility in water was rather low. The predicted Caco-2 and MDCK permeabilities for the compound under consideration exceeded 500 nm/s, indicating high intestinal permeability. The percentage of human oral absorption was also equal to 100%. The values of blood-brain permeability (QPlogBB) and human serum albumin binding constant (QPlogKhsa) are within an acceptable range, while the number of metabolic reactions is below the acceptable limit. According to the QikProp assessment, germacrene D shows excellent drug-likeness. The most important finding was that the QPlogHERG values were higher than the critical limit (−5), implying low cardiotoxic effects.

## 3. Discussion

The present study demonstrated that the essential oil of *V. nobilis* is rich in sesquiterpenes, with germacrene D identified as the predominant constituent. The high abundance of germacrene D is noteworthy because sesquiterpenes are widely recognized for their broad-spectrum antibacterial and antifungal activities [13]. Although the essential oil showed only modest antimicrobial activity, isolated germacrene D exhibited substantially stronger activity against both bacteria and fungi, particularly multidrug-resistant strains. This finding is consistent with previous reports that isolated terpenes can show greater antimicrobial activity than crude essential oils, possibly because of more effective interactions with microbial membranes and the absence of antagonistic effects from other oil constituents [14].

The antimicrobial activity of the complete EO is likely to reflect the combined effects of its constituents rather than the activity of germacrene D alone. Most constituents of *V. nobilis* oil occurred at concentrations below 1%, and their individual contributions may therefore be limited. Nevertheless, some minor constituents have been associated with antimicrobial activity. α-Cadinene, a hydrocarbon sesquiterpene, has been identified in antibacterial essential oils, including that of *Cistus creticus*, although its specific contribution to the activity of the complete oil is difficult to determine because it occurs together with other bioactive constituents [15,16]. Guaiol acetate has also been reported in essential oils with antimicrobial potential, but direct evidence for activity of the isolated compound remains limited. In contrast, stronger evidence is available for τ-cadinol, which has demonstrated antibacterial activity against *S. aureus* and has been identified, together with β-eudesmol and α-cadinol, as a contributor to the antibacterial activity of *Phoebe formosana* leaf EO. τ-Cadinol has also shown antifungal activity against several fungal species [17]. These observations are consistent with the view that oxygenated sesquiterpenes may contribute to the antimicrobial activity of essential oils [18].

Although most constituents identified in *V. nobilis* oil are hydrocarbon terpenes, nine oxygenated terpenes were also detected and may contribute to the observed activity. Oxygenated terpenes often exhibit greater antimicrobial activity than hydrocarbon terpenes because their functional groups can facilitate interactions with membrane phospholipids, proteins, and other cellular components. In contrast, highly lipophilic hydrocarbons such as limonene, camphene, α-humulene, germacrene D, δ-cadinene, and trans-caryophyllene can partition into microbial membranes, alter membrane fluidity and permeability, and promote leakage of intracellular constituents, although their lack of polar functional groups may limit interactions with specific cellular targets [18,19,20]. Oxygenated monoterpenes such as linalool and oxygenated sesquiterpenes have likewise been reported to contribute substantially to antimicrobial activity [20,21]. However, antimicrobial activity cannot be predicted solely from the presence or absence of oxygen-containing functional groups. Molecular structure, the type and position of functional groups, concentration, target microorganism, and interactions among constituents can all influence the observed activity [20,21]. Additive, synergistic, or antagonistic interactions may therefore cause the activity of the complete essential oil to differ substantially from that of individual components [15].

The markedly greater activity of germacrene D than the whole EO, despite germacrene D constituting approximately 65% of the oil, further illustrates the importance of constituent interactions. EO activity is not necessarily proportional to the abundance of its major constituent because the overall effect depends on the intrinsic potency and interactions of all components. The remaining constituents may dilute the effective concentration of germacrene D or interact with it either synergistically or antagonistically [15,21]. In the present study, the approximately twentyfold lower MIC values of germacrene D against *S. aureus* MDR 1 and *S. aureus* MDR 2 indicate substantially greater intrinsic activity than that of the complete oil. Differences in physicochemical properties, including solubility, membrane partitioning, diffusion, and stability, may also affect the availability of germacrene D to microbial cells when it is present within the complex oil matrix [20,21]. Thus, a high abundance of a constituent does not necessarily translate into equivalent biological potency, and the antimicrobial activity of *V. nobilis* oil is likely to result from the collective effects and interactions of its constituents.

The antimicrobial activity of germacrene D is consistent with the membrane-disruptive effects reported for hydrophobic sesquiterpenoids. Their lipophilic nature facilitates interaction with membrane phospholipids, which can increase membrane permeability, promote leakage of cellular components, disrupt membrane potential, impair respiratory enzymes and ATP production, and ultimately cause cell death [22,23,24,25,26,27]. Notably, germacrene D retained activity against multidrug-resistant bacteria despite their adaptive mechanisms, including reduced membrane permeability and active efflux pumps. This activity may reflect the ability of membrane-active phytochemicals to affect multiple cellular processes, potentially making them less vulnerable to existing resistance mechanisms [22,23,24].

Germacrene D exhibited bactericidal effect against most of the tested bacterial strains, suggesting that it can eliminate susceptible microbial cells rather than merely inhibit their growth. This property is particularly relevant in the context of increasing antimicrobial resistance [25]. However, these findings should be interpreted cautiously because the distinction between inhibitory and lethal effects was assessed using the agar-plug method. This approach involves excising plugs from the ZOI and re-inoculating them onto fresh culture media as a preliminary means of differentiating bacteriostatic/fungistatic from bactericidal/fungicidal activity [28,29]. Residual antimicrobial compounds retained in the agar plug may continue to inhibit microbial growth after transfer and could therefore produce a false indication of bactericidal or fungicidal activity. In addition, agar desiccation during storage may reduce microbial viability and confound interpretation [30]. The observed bactericidal and fungicidal effects should therefore be regarded as preliminary and, where possible, confirmed using quantitative methods such as viable-count or time-kill assays [29,31].

Germacrene D also showed pronounced antifungal activity, with fungicidal effects observed against all tested fungi. Although griseofulvin produced larger inhibition zones in the agar-diffusion assay, germacrene D showed lower MIC values. This apparent discrepancy highlights an important limitation of agar diffusion for hydrophobic compounds, which may diffuse poorly through agar and consequently produce smaller inhibition zones despite substantial antimicrobial potency [32,33,34]. MIC values may therefore provide a more reliable measure of the intrinsic activity of hydrophobic sesquiterpenes such as germacrene D. The antifungal activity of germacrene D may also involve disruption of fungal membrane integrity through interference with ergosterol biosynthesis [32,34]. Ergosterol is essential for maintaining fungal membrane structure and function, and inhibition of CYP51-mediated sterol 14α-demethylation is an established mechanism of action of several antifungal agents [35,36]. However, the specific mechanism of action of germacrene D against the tested fungi remains to be established.

Molecular docking of germacrene D against two clinically relevant enzymes: dehydrosqualene synthase (CrtM) from *S. aureus* and homology-modeled sterol 14-α-demethylase (CYP51) from *P. funiculosum* revealed favorable binding to both targets, with stronger affinity toward CrtM (Glide score: −7.654 kcal/mol) than CYP51 (−5.898 kcal/mol). These findings provide preliminary insights into a possible antimicrobial mechanism, although experimental validation is needed to confirm this hypothesis.

The binding to CrtM, which catalyzes the first committed step in staphyloxanthin biosynthesis, was primarily driven by hydrophobic interactions, consistent with the generally lipophilic nature of sesquiterpenes. This interaction is particularly relevant because staphyloxanthin is an important virulence-associated carotenoid pigment in *S. aureus* that contributes to oxidative-stress resistance [37,38,39]. Although germacrene D (−7.654 kcal/mol) scored lower than the native ligand ZYM (−12.265 kcal/mol), it occupied a similar active-site region, supporting potential competitive inhibition. Although the binding affinity of germacrene D (−7.654 kcal/mol) was considerably lower than that of the native ligand ZYM (−12.265 kcal/mol), which benefits from additional hydrogen bonds and π-π stacking interactions, the Glide score still indicates thermodynamically favorable binding [40]. Moreover, germacrene D exhibited antibacterial activity with MIC values as low as 10 µg/mL against the MDR *S. aureus* strains, suggests that factors beyond binding affinity—including membrane disruption, cellular uptake, and multi-target interactions—likely contribute to its efficacy [21,23].

Likewise, its moderate binding to CYP51 (−5.898 kcal/mol) involved polar contacts with HIE307, a conserved heme-coordinating residue, alongside hydrophobic interactions, reinforcing its potential to interfere with ergosterol biosynthesis in fungi [41,42]. The moderate docking score of germacrene D against CYP51 while lower than those of clinically used azoles, should be interpreted in the context of the compound’s overall mechanism. The experimentally observed antifungal activity (MIC = 50–100 µg/mL) is likely the result of a dual mechanism involving both CYP51 inhibition and membrane disruption, a common feature of sesquiterpenes [21,34,41]. This multi-target action, combined with favorable membrane permeability, explains the potent whole-cell antifungal activity despite moderate enzyme binding affinity.

The observed in vitro antimicrobial activity, which cannot be fully explained by the docking results alone, suggests that additional factors, including membrane disruption, cellular uptake, and interactions with multiple cellular targets, may contribute to the overall efficacy of germacrene D. Essential-oil constituents can perturb microbial membranes, alter membrane permeability and potential, and affect multiple cellular processes, while their antimicrobial effects may also arise from interactions with several targets rather than a single protein [43,44]. Moreover, molecular docking provides a computational model of ligand–protein recognition and should not be interpreted as a direct quantitative predictor of cellular antimicrobial potency [42].

These findings highlight the need to integrate computational predictions with experimental validation. Future structure–activity relationship studies and chemical derivatization to introduce polar groups may enhance hydrogen bonding and inhibitory potency, while molecular dynamics simulations could assess complex stability and binding thermodynamics. Overall, germacrene D emerges as a promising candidate for multitarget antimicrobial therapy, warranting further in vitro and in vivo evaluation.

ADMET analysis supports the favorable drug-like physicochemical profile of germacrene D. The compound complied with Lipinski’s rule of five, which is generally associated with physicochemical characteristics favorable for oral drug development, although compliance alone does not establish oral bioavailability or therapeutic suitability [45]. The predicted high human intestinal absorption and favorable Caco-2 and MDCK permeability further suggest that germacrene D may possess favorable absorption and permeability characteristics, although these predictions require experimental validation [46]. The high lipophilicity of germacrene D may also facilitate partitioning into microbial membranes, a property relevant to the membrane-disruptive antimicrobial mechanisms reported for hydrophobic essential-oil constituents [47]. However, poor aqueous solubility, a common limitation of hydrophobic sesquiterpenes, may compromise dissolution and bioavailability. This challenge can be addressed through optimization or advanced formulations such as nanoencapsulation, liposomes, solid lipid nanoparticles, cyclodextrin inclusion complexes, or other lipid-based delivery systems to improve solubility, stability, and efficacy [48].

Overall, the present findings establish germacrene D as the principal antimicrobial constituent of *V. nobilis* essential oil and identify it as a promising lead molecule for antimicrobial drug discovery.

### Limitations of the Study and Future Perspective

Several limitations of the present study should be acknowledged. First, the antimicrobial evaluation was conducted using in vitro assays, which provide important evidence of antimicrobial activity but do not necessarily predict efficacy in vivo. The observed activity may be influenced by factors such as compound solubility, diffusion, stability, and interactions with the assay medium. Therefore, the antimicrobial activity of germacrene D and the essential oil requires further confirmation using complementary experimental approaches and appropriate in vivo models. Second, although the essential oil exhibited antimicrobial activity, its potential therapeutic application may be limited by the inherent complexity and variability of essential-oil composition, as well as issues related to chemical stability, toxicity, bioavailability, and batch-to-batch standardization. The substantially stronger activity observed for germacrene D compared with the crude essential oil supports further investigation of this constituent as a potential antimicrobial lead; however, its therapeutic potential cannot be established from the present findings alone. Third, the molecular docking analysis provides computational predictions of potential ligand–target interactions and should therefore be interpreted as a hypothesis-generating approach rather than definitive evidence of target engagement. In particular, the moderate docking scores obtained for CYP51 do not establish that this enzyme is a direct molecular target of germacrene D. Experimental studies, including CYP51 enzyme inhibition assays and other target-specific investigations, are required to validate the predicted interaction and clarify its contribution to the observed antifungal activity. Moreover, docking does not fully account for the dynamic nature of protein–ligand interactions, membrane permeability, intracellular concentrations, or the complex biological environment.

Finally, the study did not investigate the cytotoxicity, pharmacokinetic properties, metabolic stability, or in vivo safety of germacrene D. Future studies should therefore focus on experimentally elucidating its precise molecular mechanisms of antimicrobial action, establishing its structure–activity relationships, evaluating its cytotoxicity and safety profile, characterizing its pharmacokinetic and pharmacodynamic properties, and determining its potential synergistic or additive effects in combination with conventional antimicrobial agents. Validation in appropriate animal models of infection will also be essential to determine whether the promising in vitro activity of germacrene D can translate into meaningful therapeutic efficacy in vivo.

## 4. Materials and Methods

### 4.1. Plant Material

Leaves of *V. nobilis* were collected from the Sidama region (south-central Ethiopia), during July–August 2022. The plant material was authenticated at the National Herbarium, Addis Ababa University, where voucher specimens were deposited under accession number BP002. The collected leaves were gently washed with water, air-dried at room temperature, pulverized and hydrodistilled.

### 4.2. Chemicals

All chemicals used in this study were of analytical grade and were obtained from Central Drug House Pvt. Ltd., New Delhi, India. High-purity germacrene D was procured from IndiaMART, New Delhi, India.

### 4.3. Hydrodistillation

Hydrodistillation was carried out according to Katekar et al. [49] with slight modifications. Briefly, 500 g of powdered *V. nobilis* leaves were placed in a 5 L round-bottom flask containing 2 L of distilled water. The flask was connected to a Clevenger-type apparatus fitted with a condenser, ensuring that all joints were tightly sealed. The mixture was heated to approximately 93 °C for 3 h to facilitate distillation. The condensed EO was carefully collected from the Clevenger apparatus, dried over anhydrous sodium sulfate to remove residual moisture, and stored in amber glass at 4 °C for one week before use.

### 4.4. Gas Chromatography-Mass Spectrometry (GC/MS) Analysis

Capillary Gas chromatography-Mass spectrometry (GC-MS) analysis was carried out on a GCMS-QP2010 SE instrument (Shimadzu, Kyoto, Japan), fitted with a nonpolar column; HP5-MS type capillary column (FSSupreme-5ms, CS-Chromatography Service GmbH (Langerwehe, Germany) 30 m × 0.32 mm id; 0.25 μm film thickness). Mass spectra were scanned in the range 40–500 amu. The gas chromatographic conditions were: carrier gas hydrogen (1.15 mL/min); 200 °C (injector port temperature); oven 40 °C for 3 min isothermal; 40—150 °C at 6 °C/min; 150—320 °C at 10 °C/min and then held for 3 min. Prior to analysis, the essential oil was diluted with n-hexane in 10 : 1 ratio. The diluted essential oil (1 µL) was injected at 220 °C.

The interface temperature was 280 °C. When the GC-separated component of the essential oil reached the mass spectrometer, it was ionized by 70 eV ionization voltage with a source temperature of 200 °C. The ionized samples were accelerated and separated according to mass to charge ratio (m/z) by a quadrupole mass analyzer with a temperature of 150 °C. Finally, the ionized species were detected by a MS detector with a scanning capacity of 40—500 amu.

Standard fatty acid methyl esters (Supelco 37-Komponenten FAME-Mix) were used to calibrate the retention indexes (RIs) based on the Adams library as described by [50,51], while commercial databases [52] and laboratory’s own database were used for mass spectral comparison to identify compounds. Spectral data were also compared with published linear retention indices [53]. The relative percentage composition of the essential oil was calculated using the area normalization method.

### 4.5. In Vitro Antibacterial and Antifungal Assays

#### 4.5.1. Test Microorganisms

The test microorganisms consisted of several resistant strains obtained and characterized in the Department of Pharmaceutical Technology, Jadavpur University, India. These included Gram-positive bacteria, namely *Bacillus pumilus* *B. subtilis*, and *Staphylococcus aureus*, as well as Gram-negative bacteria such as *Escherichia coli*, *Shigella flexneri*, and *Vibrio cholerae*.

The bacterial strains were cultured on nutrient agar (NA), a general-purpose medium suitable for the growth of a broad range of microorganisms. Fungal strains were maintained on Sabouraud dextrose agar (SDA) slants, which are selective media commonly used for the isolation and maintenance of fungi due to their acidic pH and high dextrose content that favor fungal growth while inhibiting most bacteria. All microbial cultures were stored at 4 °C and subcultured prior to use in the antibacterial and antifungal assays. The five drug-resistant strains included in this study were obtained from Woodlands Hospital, Kolkata, India. The strains were subsequently tested in our laboratory for their susceptibility to commonly used antibiotics, including tetracycline, streptomycin and penicillin. Since none of the strains was inhibited at a concentration of 1000 µg/mL of these antibiotics, they were considered multidrug-resistant strains.

#### 4.5.2. Disk Diffusion Method

Antibacterial and antifungal activities were evaluated using the disc diffusion technique by comparing the zones of inhibition (ZOI) produced by the essential oils of *V. nobils* and its major compound germacrene D, with those of standard antimicrobial agents (ciprofloxacin and griseofulvin). The test samples were dissolved in 1% DMSO and further diluted to concentrations ranging from 5 to 800 µg/mL (5, 10, 25, 50, 100, 200, 400, and 800 µg/mL) for antibacterial assays and 50—1500 µg/mL (100, 200, 400, 800, and 1500 µg/mL) for antifungal assays.

For antibacterial testing, nutrient agar plates were prepared, inoculated with the test microorganisms, and allowed to dry before sterile, 6 mm paper discs impregnated with the test samples were placed on the agar surface. The plates were incubated at 37 °C for 24 h, after which the ZOIs were measured. Ciprofloxacin and 1% DMSO acted as positive and negative controls, respectively [54].

For antifungal assays, the same procedure was used with Sabouraud dextrose agar (SDA) plates. The inoculated plates were incubated at 25 °C for 72 h before measuring the ZOIs. All experiments were carried out in triplicate, and the results were expressed as the mean ± standard deviation (SD).

In the antibacterial study, the ZOIs produced by the essential oil, germacrene D, and ciprofloxacin were determined at a concentration of 200 µg/mL. In the antifungal study, a concentration of 1500 µg/mL was used, with griseofulvin serving as the positive control. These concentrations were selected because they produced measurable zones of inhibition against all the test organisms, thereby allowing the antibacterial and antifungal activities of the test substances to be evaluated.

#### 4.5.3. Determination of Minimum Inhibitory Concentration (MIC)

The cosolvency technique was used to dissolve the essential oil and germacrene D. For the antibacterial assays, the test substances were initially dissolved in a minimal volume of DMSO and subsequently diluted with water. The final concentration of DMSO was adjusted not to exceed 1%. Appropriate volumes of the stock solutions were then mixed with molten nutrient agar maintained at 40–45 °C to obtain final concentrations of 5, 10, 25, 50, 100, 200, 400, and 800 µg/mL in a total volume of 30 mL using a series of McCartney bottles. A control plate containing the maximum volume of DMSO used in the assay was included to determine whether the solvent exerted any inhibitory effect. No inhibitory effect was observed, as all test organisms exhibited normal growth on the DMSO control plates.

For antifungal assays, the same procedure was followed using SDA plates. Stock solutions were incorporated into molten SDA to obtain final concentrations of 100, 200, 400, 800, and 1500 µg/mL. The media containing the respective concentrations of the test samples were aseptically poured into pre-sterilized 70 mm Petri dishes and stored at 4 °C for 24 h to facilitate uniform distribution of the test samples. Before inoculation, he plates were dried for 2 h at 37 °C for bacterial assays and 25 °C for fungal assays. A broth microdilution inoculation method was then employed, whereby a loopful (3 mm diameter) of overnight-grown microbial culture, adjusted to 10^5^ CFU/mL, was inoculated onto each quadrant of the respective Petri dishes. The MIC was defined at the lowest concentration of the test sam0les that completely inhibited visible microbial growth [55,56].

#### 4.5.4. Determination of the Mode of Action

The mode of action of the tested samples was evaluated to determine whether they exhibited bacteriostatic/fungistatic or bactericidal/fungicidal effects. Agar plugs were aseptically removed from the ZOIs and re-inoculated onto fresh culture media. Concentrations of 200 and 1500 μg/mL were selected for the mode-of-action studies for bacterial and fungal strains, respectively, as these were the concentrations used in the corresponding disk diffusion assays. The inoculated plates were wrapped with cellophane film and kept refrigerated to minimize drying of the agar before being transferred to the appropriate incubation conditions. Following incubation, microbial growth was monitored to determine whether the viable microbial count decreased over time, indicating a bactericidal/fungicidal effect, or remained relatively stable, indicating a bacteriostatic/fungistatic effect. Microbial growth was monitored for 96 h at 37 °C for bacterial strains and 25 °C for fungal strains. The results were further confirmed using viable count assays performed at 24-h intervals with test compound concentrations of 200 μg/mL for bacteria and 1500 μg/mL for fungi [55,56].

### 4.6. In Silico Methods

#### 4.6.1. Docking Against the Antibacterial Target (Dehydrosqualene Synthase (CrtM))

The crystal structure of dehydrosqualene synthase (CrtM) from *S. aureus* (PDB ID: 4F6V, 2.30 Å), complexed with BPH-1034, Mg^2+^, farnesyl monophosphate (FMP), and the co-crystallized ligand 2,4-dioxo-4-{[3-(3-phenoxyphenyl)propyl]amino}butanoic acid (ZYM), was retrieved from the Protein Data Bank (PDB). The protein was prepared using the Protein Preparation Wizard in Schrödinger 2025–2 [40,57] with default parameters. Hydrogen addition and bond order assignment were performed, followed by removal of all but the significant crystallographic waters. Missing side chains were built with Prime [56], and their ionization states were optimized at pH 7.0 ± 2.0 using Epik [58]. Following standard procedures, the ligand was prepared using LigPrep. A receptor grid for docking was generated around the co-crystallized ligand, focusing on the binding site and including NAD. Docking was performed using the Glide module in Maestro in Standard Precision (SP) mode. Germacrene D was docked into the active-site grid box. The SP protocol uses a balanced scoring function and sampling strategy to efficiently evaluate ligand poses, providing reliable screening.

The Glide SP protocol was validated by redocking the co-crystallized (native) ligand, achieving a root-mean-square deviation (RMSD) of <2.0 Å, indicating reliable reproduction of the experimental binding pose [40].

#### 4.6.2. Docking Against the Antifungal Target (Sterol 14-α-Demethylase (CYP51)

##### Homology-Based Protein Structure Modeling

Since no experimentally determined three-dimensional structure of sterol 14-α-demethylase (CYP51) from *P. funiculosum* is available in the Protein Data Bank (PDB), a homology model was generated using the SWISS-MODEL Workspace [59]. The 515-amino-acid CYP51 protein sequence from *P. notatum* (UniProt accession number A0ABQ8W6D1) was retrieved from the UniProt database and used as the template sequence to construct a homology-modeled sterol 14α-demethylase (CYP51) from *P. funiculosum*. Suitable templates were identified using BLAST and HHblits searches against the SWISS-MODEL Template Library (SMTL) and selected based on sequence identity, coverage, and GMQE/QMEAN scores [59]. Protein structure modelling was performed using the SWISS-MODEL web server (https://swissmodel.expasy.org/; accessed on 10 June 2026). The SWISS-MODEL Template Library is a curated structural database that is continuously updated with experimentally determined structures from the Protein Data Bank. Model construction was performed using the ProMod3 modeling engine implemented in SWISS-MODEL [60].

The resulting model was evaluated using the sequence identity, Global Model Quality Estimation (GMQE), Qualitative Model Energy Analysis (QMEAN), and QMEANDisCo scores provided by SWISS-MODEL. Structural quality was further assessed using a Ramachandran plot to determine the distribution of residues in favored regions. Models showing high validation scores were considered suitable for subsequent molecular docking studies.

SWISS-MODEL was selected over alternative structure-prediction approaches, such as AlphaFold, because the target CYP51 sequence exhibited very high sequence identity (93.59%) with the available template structure from *P. notatum*, supporting the reliability of homology modeling [61]. At such high sequence identity, homology modeling can generate structures with accuracy comparable to experimentally determined models. In addition, SWISS-MODEL provides established validation metrics, including GMQE, QMEAN, QMEANDisCo, and Ramachandran analysis, enabling comprehensive assessment of model quality and reproducibility [59]. The generated model showed excellent validation statistics, with a GMQE score of 0.92 and 96.88% of residues located in favored Ramachandran regions, indicating that the model was structurally reliable and suitable for downstream molecular docking studies.

The 3D homology model of CYP51 from *P. funiculosum* was generated using the SWISS-MODEL and downloaded in PDB format and subsequently used for molecular docking studies.

##### Docking Protocol

We followed the same procedure for Protein Preparation and LigPrep as described for the antibacterial targets. Since the homology model lacked a bound ligand, the SiteMap module of the Schrödinger Suite [57] was used to predict and identify the active site. The site with the highest SiteScore (>1.0) and Dscore was selected for receptor grid generation and subsequent docking studies. Receptor grid generation was performed using the active site identified by the SiteMap module. Docking results and MM-GBSA binding free energies were evaluated and compared based on Glide docking scores, predicted binding energies, and key protein–ligand interactions, including hydrogen bonds and hydrophobic contacts [40]. The CrtM docking protocol was validated by redocking the native ligand, yielding an RMSD of <2.0 Å between the predicted and experimental binding poses. For the homology-modeled CYP51 structure, which lacked a bound ligand, the active site was identified using the SiteMap module, with a SiteScore > 1.0.

#### 4.6.3. Drug-Likeness and Pharmacokinetics Studies

The drug-likeness and pharmacokinetic properties of germacrene D were further assessed using the QikProp module [57], which was employed to predict ADMET (absorption, distribution, metabolism, excretion, and toxicity) parameters. The compound was analyzed using the default settings to estimate key physicochemical and pharmacokinetic descriptors, including the octanol/water partition coefficient (QPlogP(o/w)), aqueous solubility (QPlogS), Caco-2 cell permeability (QPPCaco2), Madin–Darby canine kidney (MDCK) cell permeability (QPPMDCK), brain/blood partition coefficient (QPlogBB), human serum albumin binding (QPlogKhsa), and the predicted inhibition of the HERG K^+^ channel (QPlogHERG, predicted log IC_50_). The predicted properties were evaluated against established drug-likeness criteria, including Lipinski’s Rule of Five, to determine the compound’s suitability as a potential drug candidate.

### 4.7. Statistical Analysis

The antimicrobial data were analyzed using SPSS (Statistical Package for the Social Sciences) software, version 26. ZOI values are presented as mean ± standard deviation (SD) from three independent determinations (*n* = 3), with the 6 mm disc diameter included in the measurements. MIC values are reported as the mean of three independent experiments, each performed in triplicate. Statistical analysis was performed on the ZOI data using one-way analysis of variance (ANOVA), followed by Tukey’s post hoc multiple-comparison test. A *p*-value < 0.05 was considered statistically significant.

## 5. Conclusions

In this study, analysis of the chemical composition of *V. nobilis* leaf essential oil revealed germacrene D as the predominant constituent, accounting for 64.75% of the total oil. Based on the MIC values, germacrene D exhibited stronger antibacterial and antifungal activities than the crude EO, with particularly pronounced activity against both MDR and non-MDR Gram-negative bacterial strains, underscoring its promising antimicrobial potential. Although the overall antimicrobial activity of the EO was lower than that of its major constituent, germacrene D, the present findings provide scientific evidence supporting the traditional use of *V. nobilis* leaves in East Africa for the treatment of respiratory tract infections. Molecular docking revealed favorable interactions of germacrene D with the bacterial CrtM and fungal homology-modeled CYP51 targets, providing preliminary insights into possible molecular interactions; however, these in silico findings do not establish a definitive mechanism of action. Similarly, the in silico ADMET analysis suggested generally favorable drug-like and pharmacokinetic properties, although poor solubility was identified as a potential limitation. Overall, the findings support germacrene D as a promising candidate for further investigation as an antimicrobial agent, while its specific molecular targets and mechanisms of action remain to be experimentally validated. Further studies should include target-validation experiments, evaluation of cytotoxicity and selectivity, detailed pharmacokinetic and bioavailability assessments, investigation of possible synergistic interactions with conventional antimicrobials, and ultimately in vivo studies to establish its antimicrobial efficacy and safety.

## Figures and Tables

**Table 1:** Chemical composition of the essential oil from the leaves of *Vepris nobilis*.

Compound	% Composition	RI Exp	RI Lit	Methods of Identification
4-Carene	0.09	915	919	RI and MS
Camphene	0.07	933	943	RI and MS
Limonene	0.06	1021	1018	RI and MS
*trans*-β-Ocimene	0.20	1043	1044	RI and MS
Linalool	0.18	1099	1095	RI and MS
Perillene	0.03	1116	1125	RI and MS
Geijerene	0.15	1144	1138	RI and MS
Pregeijerene	0.04	1288	1 285	RI and MS
δ-Elemene	0.77	1322	1335	RI and MS
α-Cubebene	0.04	1335	1345	RI and MS
1-Methyl-3-(vinyloxy)-1-cyclohexene	0.03	1340	-NA	MS
Longicyclene	0.14	1360	1371	RI and MS
α-Copaene	0.29	1370	1374	RI and MS
β-Bourbonene	0.19	1380	1387	RI and MS
β-Cubebene	0.16	1386	1387	RI and MS
β-Elemene	0.30	1389	1389	RI and MS
α-Gurjunene	0.01	1413	1409	RI and MS
*trans*-Caryophyllene	0.42	1428	1417	RI and MS
β-Copaene	0.09	1438	1430	RI and MS
α-Humulene	0.38	1464	1452	RI and MS
Alloaromadendrene	0.29	1471	1458	RI and MS
Germacrene D	64.75	1491	1484	RI and MS
Bicyclogermacrene	1.04	1499	1500	RI and MS
(*trans*, *trans*)-α-Farnesene	0.61	1507	1505	RI and MS
δ-Cadinene	3.18	1533	1522	RI and MS
Elemol	0.14	1560	1548	RI and MS
τ-Cadinol	2.38	1582	1580	RI and MS
Cubenol	1.18	1660	1645	RI and MS
Eudesm-7(11)-en-4-ol	1.18	1700	1700	RI and MS
α-Bisabolol	0.70	1678	1685	RI and MS
Guaiol acetate	3.48	1732	1725	RI and MS
Nonadecane	0.81	1900	1900	RI and MS
(*cis/trans*)-Geranyl linalool	0.83	2010	1988	RI and MS
Phytol	1.08	2080	2111	RI and MS
Docosane	0.64	2200	2200	RI and MS
Triacontane	1.00	3007	3007	RI and MS
Dotriacontane	0.55	3200	3200	RI and MS
**Total**	**87.48**			

Note: RI Exp: Experimentally measured retention index (RI) values; RI Lit: Retention index (RI) values obtained from the literature; MS: Mass spectrum; NA: Not available; Percentage composition represents each compound’s relative contribution to the total integrated peak area (normalization method).

**Table 2:** Antibacterial activity of *Vepris nobilis* leaf essential oil and its major constituent, germacrene D, at a concentration of 200 μg/mL against selected bacterial strains.

Bacterial Strain	*V. nobilis* EO	Germacrene D	Ciprofloxacin
*Bacillus pumilus* 82	8.8 ± 0.3 ^c^ (400)	9.5 ± 0.0 ^c^ (50)	19.0 ± 0.2 ^a^ (100)
*B. subtilis*	8.7 ± 0.3 ^c^ (400)	9.5 ± 0.0 ^c^ (50)	18.0 ± 0.2 ^a^ (100)
*Escherichia coli* 3:37C	12.5 ± 0.0 ^b^ (25)	14.5 ± 0.5 ^a^ (10)	16.5 ± 0.0 ^a^ (25)
*E. coli* 872	12.5 ± 0.0 ^c^ (25)	15.0 ± 0.0 ^a^ (10)	16.0 ± 0.3 ^a^ (25)
*E. coli* C600**	12.0 ± 0.0 ^c^ (25)	14.0 ± 0.0 ^a^ (10)	13.5 ± 0.2 ^ab^ (25)
*E. coli* CD/99/1	13.0 ± 0.5 ^c^ (25)	14.5 ± 0.0 ^bc^ (10)	17.0 ± 0.2 ^a^ (25)
*E. coli* HB101 **	13.0 ± 0.0 ^b^ (25)	14.0 ± 0.0 ^ab^ (10)	14.0 ± 0.3 ^a^ (25)
*E. coli* K88	12.5 ± 0.5 ^c^ (25)	15.0 ± 0.0 ^ab^ (10)	17.1 ± 0.2 ^a^ (25)
*E. coli* LT37	12.5 ± 0.0 ^b^ (25)	14.5 ± 0.5 ^a^ (10)	16.0 ± 0.0 ^a^ (10)
*E. coli* NCTC 5933	12.0 ± 0.0 ^c^ (25)	14.0 ± 0.0 ^ab^ (10)	16.0 ± 0.3 ^a^ (25)
*E. coli* NCTC 7360	13.5 ± 0.5 ^b^ (25)	15.0 ± 0.0 ^a^ (10)	17.0 ± 0.2 ^a^ (25)
*E. coli* ROW 7/12	12.5 ± 0.0 ^c^ (25)	15.5 ± 0.5 ^ab^ (10)	16.5 ± 0.0 ^a^ (25)
*Pseudomonas aeruginosa* MDR 1 **	11.0 ± 0.5 ^b^ (100)	12.0 ± 0.0 ^ab^ (10)	12.5 ± 0.1 ^a^ (50)
*Staphylococcus aureus* MDR 1 **	10.3 ± 0.3 ^c^ (200)	13.0 ± 0.5 ^a^ (10)	11.5 ± 0.3 ^b^ (200)
*S. aureus* MDR 2 **	10.2 ± 0.3 ^c^ (200)	13.5 ± 0.0 ^a^ (10)	12.0 ± 0.0 ^b^ (200)
*S. aureus* ML 267	17.0 ± 0.0 ^b^ (10)	19.0 ± 0.5 ^a^ (50)	18.0 ± 0.2 ^ab^ (50)
*Salmonella enterica* TD 01	15.0 ± 0.0 ^b^ (25)	14.5 ± 0.5 ^b^ (50)	19.0 ± 0.1 ^a^ (150)
*S. typhi* Ty2	14.0 ± 0.0 ^b^ (25)	14.5 ± 0.5 ^ab^ (50)	16.0 ± 0.2 ^a^ (200)
*Shigella boydii*	13.5 ± 0.5 ^b^ (25)	14.5 ± 0.5 ^ab^ (50)	20.0 ± 0.0 ^a^ (150)
*S. dysentery* 8	15.0 ± 0.5 ^b^ (25)	16.3 ± 0.5 ^ab^ (50)	20.0 ± 0.1 ^a^ (150)
*S. flexneri*	13.0 ± 0.0 ^b^ (25)	15.0 ± 0.0 ^ab^ (50)	20.5 ± 0.0 ^a^ (150)
*S. soneii* 1	15.0 ± 0.0 ^b^ (25)	16.2 ± 0.8 ^ab^ (50)	19.5 ± 0.0 ^a^ (150)
*Vibro cholerae* NCTC 10732	15.0 ± 0.0 ^ab^ (25)	15.0 ± 0.0 ^ab^ (25)	19.0 ± 0.2 ^a^ (25)
*V. cholerae* NCTC 11501	12.0 ± 0.5 ^b^ (25)	14.0 ± 0.5 ^ab^ (25)	18.5 ± 0.2 ^a^ (25)
*V. cholerae* NCTC 4693	14.0 ± 0.0 ^b^ (25)	14.0 ± 0.0 ^b^ (25)	17.5 ± 0.0 ^a^ (25)
*V. cholerae* NCTC 5596	14.5 ± 0.0 ^ab^ (25)	15.0 ± 0.5 ^ab^ (50)	18.5 ± 0.1 ^a^ (25)

Antibacterial activity is expressed as zone of inhibition (ZoI, mm) presented as mean ± standard deviation (SD) (*n* = 3). Values in brackets represent minimum inhibitory concentrations in µg/mL. MIC values represent the mean of three independent experiments performed in triplicate. Within each row, means followed by different superscript letters (a–c) are significantly different at *p* < 0.05, as determined by one-way ANOVA followed by Tukey’s post hoc test. ** Multidrug resistant (MDR) strains.

**Table 3:** Antifungal activities of *Vepris nobilis* leaf essential oil and its major constituent, germacrene D, at a concentration of 1500 μg/mL against selected fungal strains.

Fungal Strain	*V. nobilis*	Germacrene D	Griseofulvin
*Aspergillus niger*	12.50 ± 0.00 ^c^ (400)	13.33 ± 0.29 ^b^ (50)	14.54 ± 0.39 ^a^ (400)
*Candida albicans*	11.67 ± 0.29 ^d^ (400)	14.50 ± 0.00 ^b^ (50)	15.17 ± 0.29 ^a^ (500)
*Penicillium notatum*	14.00 ± 0.50 ^a^ (400)	13.00 ± 0.00 ^b^ (100)	12.03 ± 0.75 ^c^ (500)
*Penicillium funiculosum*	14.00 ± 0.00 ^a^ (400)	13.00 ± 0.00 ^b^ (50)	11.00 ± 0.29 ^c^ (500)

Antifungal activity is expressed as zone of inhibition (ZoI, mm) presented as mean ± standard deviation (SD) (*n* = 3); Values in brackets represent minimum inhibitory concentrations (MICs) in µg/mL; MIC values represent the mean of three independent experiments performed in triplicate; Within each row, means followed by different superscript letters (a–d) are significantly different at *p* < 0.05, as determined by one-way ANOVA followed by Tukey’s post hoc test.

**Table 4:** Structural Validation for the Homology-Modeled CYP51 Protein of *Penicillium funiculosum* Generated via SWISS-MODEL.

Template Protein	Template UniProt Accession Number	GMQE	QMEAN Score	Sequence Identity (%)	Alignment Coverage	Template Resolution	QMEANDisCo Global Score	Ramachandran Plot
Sterol 14-α-demethylase (CYP51) of *Penicillium notatum*	A0ABQ8W6D1	0.92	0.43	93.59%	1.00	−1.00	0.81 ± 0.05	96.88%

**Table 5:** Standard-precision (SP) Glide docking scores for germacrene D with *Staphylococcus aureus* dehydrosqualene synthase (CrtM; PDB: 4F6V).

Compounds	G.Score (kcal/mol)	Interacting Amino Acids	Types of Interaction
Germacrene D	−7.654	ALA134; ALA157; ALA244; ALA245; GLY138; GLY161; ILE241; LEU141; LEU145; LEU160; LEU164; PHE233; PHE22; PHE26; THR142; VAL137	Polar interaction (THR142) Hydrophobic interaction with the rest of the interacting amino acids, except GLY138 and GLY161
Native ligand 2,4-dioxo-4-{[3-(3-phenoxyphenyl) propyl]amino}butanoic acid (ZYM)	−12.265	ALA134; ARG45; ARG171; ARG265; ASN168; ASP48; ASP172; GLN165; ILE40; LEU107; MET15; PHE22; PHE26; TYR41; VAL37; VAL135	Pi-Pi stacking (TYR141), Hydrogen bonding (ASP48, ASN168, ARG265), Hydrophobic interaction (MET15, PHE22, PHE26, VAL37, ILE40, ARG45)

**Table 6:** Standard-precision (SP) Glide binding scores for germacrene D with homology-modeled structure of *Penicillium funiculosum* sterol 14α-demethylase.

Compound	G.Score (kcal/mol)	Interacting Amino Acids	Types of Interaction
Germacrene D	−5.898	ALA304; CYS459; HIE307; ILE370; LEU373; LEU499; MET303; PHE120; PHE500; TYR112; THR116; TYR126; PHE219; THR300; VAL125	Polar interaction (THR116; THR300; HIE307); Hydrophobic interaction with the rest of the interacting amino acids.

**Table 7:** QikProp drug-likeness and ADME descriptors for germacrene D.

Compound	M. Weight	QPlogP (o/w)	QPlogS	QPPCaco2	QPlogBB	QPPMDCK	QPlogKhsa	#Metab	QPlogHERG	Rule of Five	Percent Human Oral Absorption
Germacrene D	208.386	−0.459	−6.846	9906.038	1.156	5899.293	1.094	2	−3.239	1	100.00
Recommended values	<500	−2.0 to 6.5	−6.5 to 0.5	>500	−3.0 to 1.2	>500	−1.5 to 1.5	1 to 8	≥−5	≤1	>80%

## Data Availability

The data that support the findings of this study are available in the Supplementary Materials of this article. If there are more requirements, the data are available from the corresponding author upon reasonable request.

## Supplementary Materials

The following supporting information can be downloaded at: https://www.mdpi.com/article/10.3390/ijms27177927/s1.
