# Supplementary material for: Chemical Profiling and Antimicrobial Activity of the Leaf Essential Oil of Vepris nobilis (Delile) Mziray: In Silico Evaluation of the Major Constituent, Germacrene D
Source: Int J Mol Sci. 2026 Sep 5;27(17):7927. doi: 10.3390/ijms27177927 (PMC13566347; doi:10.3390/ijms27177927)
Supplement: Supplementary file 1 [file ijms-27-07927-s001.zip › ijms-4502313-supplementary.pdf]

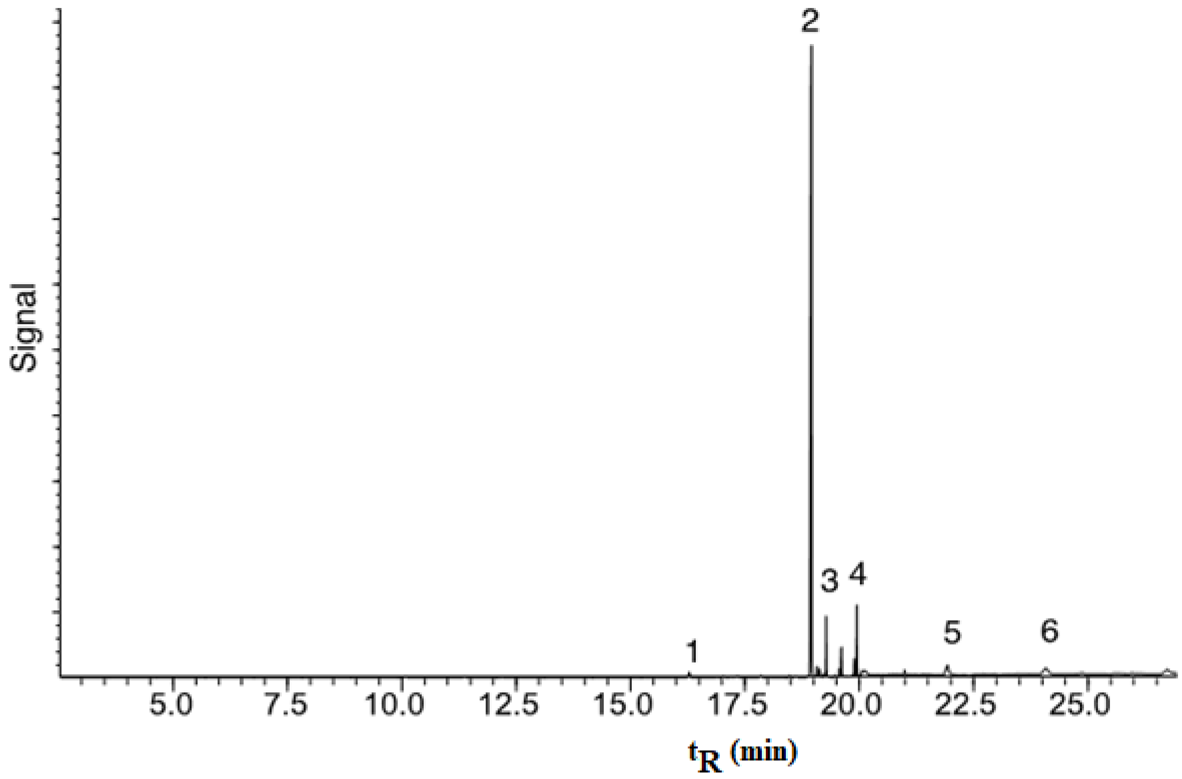

Figure S1. The GC chromatogram of the essential oil from *Vepris nobilis* (**1**: δ-Elemene; **2**: Germacrene D; **3**: δ-Cadinene; **4**: Guaiol acetate; **5**: Unidentified; **6**: Unidentified).
